# A New Framework Combining Diffusion Models and the Convolution Classifier for Generating Images from EEG Signals

**DOI:** 10.3390/brainsci14050478

**Published:** 2024-05-08

**Authors:** Guangyu Yang, Jinguo Liu

**Affiliations:** 1 State Key Laboratory of Robotics, Shenyang Institute of Automation, Chinese Academy of Sciences, Shenyang 110016, China; yangguangyu1@sia.cn; 2 University of Chinese Academy of Sciences, Beijing 100049, China

**Keywords:** electroencephalography, brain–computer interface, image generation, stable diffusion, convolutional neural network

## Abstract

The generation of images from electroencephalography (EEG) signals has become a popular research topic in recent research because it can bridge the gap between brain signals and visual stimuli and has wide application prospects in neuroscience and computer vision. However, due to the high complexity of EEG signals, the reconstruction of visual stimuli through EEG signals continues to pose a challenge. In this work, we propose an EEG-ConDiffusion framework that involves three stages: feature extraction, fine-tuning of the pretrained model, and image generation. In the EEG-ConDiffusion framework, classification features of EEG signals are first obtained through the feature extraction block. Then, the classification features are taken as conditions to fine-tune the stable diffusion model in the image generation block to generate images with corresponding semantics. This framework combines EEG classification and image generation means to enhance the quality of generated images. Our proposed framework was tested on an EEG-based visual classification dataset. The performance of our framework is measured by classification accuracy, 50-way top-k accuracy, and inception score. The results indicate that the proposed EEG-Condiffusion framework can extract effective classification features and generate high-quality images from EEG signals to realize EEG-to-image conversion.

## 1. Introduction

Decoding the correlation between brain signals and human visual perception has grown to be a research focus. It can promote the progress of cognitive neuroscience and expand the application of brain signals. One of the most popular methods is to reconstruct images from brain signals [1,2,3].

Brain signals can be obtained through both invasive and noninvasive methods. Noninvasive methods have attracted much attention because of their advantages of high security, high acceptance by users, and wide application [4]. The electroencephalogram (EEG) is an important non-invasive brain signal, as well as functional magnetic resonance imaging (fMRI) [5]. In recent years, owing to the consistency of the structure of fMRI and image data as visual stimuli, many scholars have attempted to reconstruct images from fMRI [6,7,8,9,10]. However, owing to the large size of the fMRI equipment and the high level of professional skills needed, its application scope is limited. On the other hand, EEG equipment that can record signals wirelessly, is more portable, and is easier to operate, which reduces the cost and difficulty of signal collection [11]. In addition, research in [12] has shown that brain signals recorded by EEG can be used to decode visual stimuli. Therefore, methods that can efficiently extract EEG features and reconstruct high-quality images urgently need to be studied.

Currently, deep learning methods are broadly adopted in EEG decoding. Recurrent neural networks (RNNs) and convolutional neural networks (CNNs) are two established deep learning structures. They not only can be trained end-to-end but can also be easily combined with popular large artificial intelligence models, expanding the scope of EEG applications. Spampinato et al. [12] recorded the EEG dataset of six subjects while watching 40 classes of images from the ImageNet dataset [13]. The long short-term memory (LSTM) model [14] was employed to recognize the EEG signals. However, LSTM can usually only process information in a single dimension, making it tough to understand the spatio–temporal semantic information of EEG signals in detail. Palazzo et al. [1] processed EEG information of different dimensions by designing time blocks and space blocks in layers and verified the performance on the dataset [12]. Lawhern et al. combined the ideas of CNNs and FBCSP [15] to propose a compact CNN structure called EEGNet [16]. EEGNet can extract features of different dimensions simultaneously through two-step convolution. In addition, good decoding accuracy has been achieved on the EEG signals of various paradigms, indicating that the EEGNet has good robustness and generalizability.

EEG image generation methods have developed rapidly in recent years. The most mainstream methods include generative adversarial networks (GANs) [17] together with variational autoencoders (VAEs) [18]. Diffusion models are new developments [19,20,21,22,23,24]. A VAE is a generative model based on variational inference, and its data distribution and loss function tend to produce unrealistic and fuzzy samples. Kavasidis et al. [25,26] proposed the Brain2Image method. They used extracted EEG features as a manifold to guide the training of a GAN, generated more realistic images, and compared the effects of VAEs and GANs on image reconstruction from EEG signals. We commonly employ the inception score as a metric to assess the quality of generated images. Additionally, the results of VAE [25] and GAN [25] are 4.49 and 5.07, respectively. They found that although images generated by the GAN were not very realistic, they were clearer than those generated by the VAE. Zheng et al. [27] applied a combined LSTM–CNN structure to extract EEG features and adopted an improved spectral normalization generative adversarial network (SNGAN) [28] for image generation. Khare et al. [29] proposed a NeuroVision architecture that utilizes EEG classification features to guide the training of progressive growth of GANs (ProGAN) [30] to improve image generation performance. Additionally, the inception results of SNGAN [27] and cProGAN [29] are 5.53, and 5.15, respectively. GANs generate higher-resolution images via adversarial learning. However, the training of a GAN can be unstable and prone to problems such as mode crashes and training oscillations. Compared to GANs, diffusion models avoid the problem of mode collapse and can simulate more complex data distributions. In [22], it was confirmed that diffusion models can generate images with higher resolutions than a GAN or VAE. However, training a diffusion model from scratch requires not only a large number of EEG–image data pairs but also significant computational resources. Therefore, we consider finding a suitable pretraining model to solve this problem. Recently, methods based on diffusion models have made significant breakthroughs in text-to-image generation [31,32,33,34], achieving the conversion of different modality signals. In addition, a latent text-to-image diffusion model was jointly developed by Stability AI and LAION based on [24]. This process was named stable diffusion (SD). SD has developed rapidly in the field of AI painting and generation because of its efficient and stable generation capabilities, simple model optimization, and portability [35,36,37]. This has also inspired research on generating images from brain signals. Some scholars have combined the SD model with tasks of generating images from fMRI and obtained high-quality reconstructed images [10,38,39]. They demonstrated the feasibility of the SD model in brain-to-image tasks.

In this work, we intend to realize the EEG-to-image task by utilizing the powerful generative ability of a pretrained SD model. However, on the one hand, EEG signals have low spatial resolution and a low signal-to-noise ratio, and their distribution space is different from that of images. On the other hand, the SD model is trained based on text–image data, and it is difficult to obtain good results when the model is directly applied to EEG-to-image tasks. To address these challenges, we propose a comprehensive EEG-ConDiffusion framework for generating corresponding images from EEG signals. The EEG signal passed through the framework first passes through a feature extraction block, which obtains effective input features. The feature extraction block is centered on convolutional neural networks, combining the advantages of EEGNet and residual networks. It can not only extract features from EEG data in both temporal and spatial dimensions but also avoid problems such as gradient explosion, overfitting, and decreased learning ability, which arise from deep networks. Then, we perform position encoding and shape transformation on the obtained EEG features to adapt to the input format of the SD model. Finally, we use EEG features and image pairs to fine-tune the SD model. We integrate the EEG classification task with the image generation task and use features with obvious semantic distinctions to generate images. The principal contributions of our work are outlined below:

- We designed a framework called EEG-ConDiffusion for generating images from EEG signals. It integrates the powerful feature extraction capabilities of CNNs and the image generation capabilities of the SD model to achieve decoding from EEG signals to images. In addition, our framework has been validated on an EEG-based visual classification dataset [12].
- We propose a convolutional neural network for EEG feature extraction. We design temporal convolutional layers and spatial convolutional layers separately to achieve multi-dimensional, comprehensive extraction of EEG features. At the same time, we introduce multiple residual blocks to improve network depth while avoiding problems such as gradient explosion and overfitting.
- We use the features extracted through the positional encoding network to change the size of the input features to adapt to the input of the SD model. In addition, the distribution of the input features more closely matches the text.
- In addition, we use processed EEG features and image data to fine-tune the stable diffusion model, making it suitable for EEG-to-image tasks. Then, the extracted EEG features are fed into the SD model with updated training parameters to complete the image generation task.

Section 2 introduces the main components of EEG-ConDiffusion and the corresponding implementation methods. In Section 3, we tested the proposed EEG-ConDiffusion method on a public dataset [12] and displayed the results. In Section 4, we discuss the validation results for each stage. Additionally, we summarize the whole paper in Section 5.

## 2. Materials and Methods

This section presents the dataset used for experimental verification in this paper. In addition, the various components of the proposed EEG-ConDiffusion framework are presented and explained in detail.

### 2.1. The Public Dataset

We conducted performance verification on the EEG-based visual classification dataset [12]. The dataset recorded 12,000 trials of EEG data from six subjects as they viewed visual stimuli. The dataset was recorded using 128-electrode Brainvision EEG equipment with a sampling frequency of 1000 Hz. A total of 2000 images were used as visual stimuli, including 50 for each of the 40 different categories in the ImageNet dataset [13]. Each image was rendered for 0.5 s.

We adopted the same data preprocessing method as [16] and selected 20–460 ms of EEG data. Therefore, the EEG data shape for each trial was set (1,128,440). The dataset was divided into 70%, 15%, and 15% ratios, which were utilized for training, validation, and testing of experiments, respectively. Furthermore, we separately filtered the signals in the frequency ranges of 1–70 Hz and 5–95 Hz for subsequent model evaluation.

### 2.2. The Proposed EEG-ConDiffusion Framework

**Figure 1:**
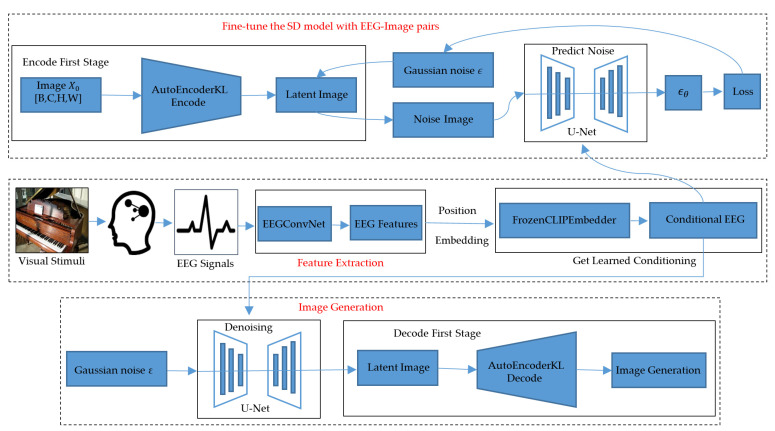
The overall structure of the proposed EEG-ConDiffusion framework. $\varepsilon_{\theta }$ represents the output noise of the diffusion model.

Figure 1 shows the overall structure of our proposed EEG-ConDiffusion framework. Our EEG-ConDiffusion framework is mainly composed of three stages: feature extraction, fine-tuning of the SD model, and image generation. EEG signals are extracted by a convolutional neural network model, which we call EEGConvNet. With the EEGConvNet model, we can obtain highly distinguishing class features. We use the category features as the guiding conditions of the SD model, so as to ensure the authenticity of the generated images. The EEG features are processed as the embedding input of the SD model through a position embedding network and a pretrained text encoder, FrozenCLIPEmbedder. We refer to this embedding input as conditional EEG (ConEEG). ConEEG was used as a condition to input the SD model through U-Net, and then model fine-tuning and image generation were performed.

#### 2.2.1. Feature Extraction

**Figure 2:**
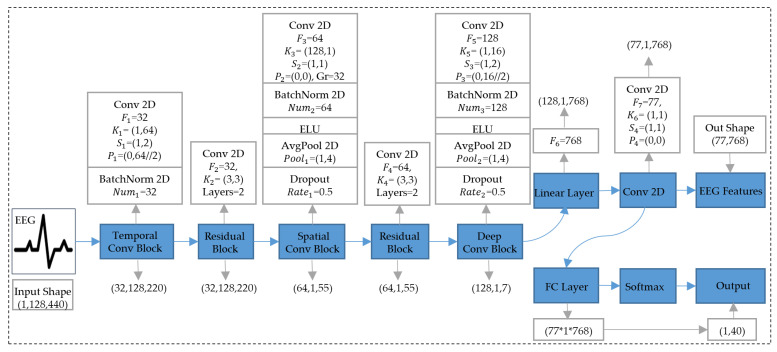
The structure and parameters of the EEGConvNet model. $F_{1}∽F_{5}$ represent the out_channels for the different Conv2d layers. $F_{6}$ indicates the out_features of different linear layers. $K_{1}∽K_{6}$, $S_{1}∽S_{4}$, and $P_{1}∽P_{4}$ represent the kernel_size, stride, and padding of different Conv2d layers, respectively. ${Num}_{1}$ and ${Num}_{2}$ represent num_features of different BatchNorm2d layers. ${Rate}_{1}$ and ${Rate}_{2}$ are used to refer to the deactivation probabilities P for different Dropout layers. Layers represent the number of residual blocks.

Extracting highly discriminative semantic features from EEG signals is a key step in realizing EEG-to-image. Hence, we designed a CNN structure called EEGConvNet for EEG feature extraction. As shown in Figure 2, the EEGConvNet model is composed of a temporal convolution (TC) block, two residual (Res) blocks, a spatial convolution (SC) block, a deep convolution (DC) block, and two-branch output networks.

Inspired by EEGNet [16], we jointly use TC and SC blocks to better extract the temporal and spatial information of EEG signals. For the TC block, we set the parameter of out_channels to 32 and the stride to (1,2) in the Conv2d layer. The parameters of the out_channels correspond to the width of the network. Increasing the stride can reduce the amount of computation and quickly obtain important information. For the SC block, we also increased the number of out_channels in Conv2d to 64. In the DC block, we set the out_channels parameter to 128 and the stride parameter to (1,2). To avoid semantic confusion and gradient explosion caused by deepening the network and to further extract richer semantic information from the EEG, we introduce residual networks [40] in EEGConvNet. Two double-layer residual blocks are inserted between the TC block, SC block, and DC block.

In addition, the EEG features output by the DC block pass through a linear layer and a Conv2d layer, and the squeeze function is used to reduce the dimension. Thus, the output EEG feature shape is processed as (77,768), which can serve as the input of the SD model. Our EEGConvNet structure has two output branches. One to output the EEG features for the SD model and the other to output classification results.

When training the EEGConvNet model, the input shape size of the EEG data was 1,128,440. After passing through the TC block, the SC block, residual blocks, and the DC block, the output shape of the EEG eigenvector is (128,1,7). Then, the EEG eigenvector is further processed by a linear layer and a Conv2d layer with a kernel size of (1,1) to obtain the shape output (77,1,768). Furthermore, the EEG feature vector (77,768) and the classification vector (1,40) were output from the EEG data through two branches. In addition, we apply operations such as BatchNorm, dropout, and average pooling in the EEGConvNet model and use ReLU as the activation function. These layers can expand the receptive field of the model and reduce overfitting.

#### 2.2.2. Fine-Tuning the SD Model with EEG–Image Pairs

Diffusion models are currently one of the hottest directions in the research of artificial intelligence-generated content (AIGC). The SD model is a text-to-image model developed based on latent diffusion models (LDMs) proposed by Rombach et al. [24]. LDMs perform the diffusion process on the latent space, which greatly reduces the computational complexity and cost. In addition, the cross-attention method is proposed for multimodal training, and the task of multimodal conditional generation is realized. In this paper, we input the EEG features that passed through the feature extraction stage as a conditional input into the cross-attention module in U-Net to guide the training of the model.

Jonathan et al. [19] were the first to provide a rigorous mathematical derivation of diffusion models (DMs) and established a complete framework for the forward process, reverse process, and model training. The positive diffusion propagation can be regarded as a Markov process. Given an image $x_{0}$, $x_{0}\in R^{H\times W\times 3}$, the distribution of the natural image is defined as $x_{0}∽q\left(x_{0}\right)$. In the forward process, Gaussian noise is gradually added to the input $x_{0}$, resulting in multiple noisy samples $x_{1}$, $x_{2}$…, $x_{T}$. The variance of the added noise is a fixed value $\beta_{t}\epsilon \left(0,1\right)$. The mean value of the noise is determined by $\beta_{t}$ and $x_{t}$. After the T noise operation, image $x_{T}$ becomes a pure noise image that conforms to the standard normal distribution. The forward process can be represented as follows:

(1) $$q\left(x_{1}:x_{T}|x_{0}\right)\;∶=\prod_{t=1}^{T}q\left(x_{t}|x_{t-1}\right)$$

The mathematical description of the conditional probability distribution of the process is as follows:

(2) $$q\left(x_{t}|x_{t-1}\right)\;∶=N\left(x_{t};\sqrt{1-\beta_{t}}x_{t-1},\beta_{t}Ι\right)$$

The reverse process is to denoise from the Gaussian noise and reconstruct the original data, which is also a Markov process. In this process, we need to construct a parameter distribution to make an estimate. The mean and variance of the predicted noise are determined by the input time t and the current image $x_{t}$. The process can be expressed as:

(3) $$p_{\theta }\left(x_{0}:x_{T}\right)\;∶=p\left(x_{T}\right)\prod_{t=1}^{T}p_{\theta }\left(x_{t-1}|x_{t}\right)$$

The conditional probability distribution for the reverse process is expressed as follows:

(4) $$p_{\theta }\left(x_{t-1}|x_{t}\right)\;∶=N\left(x_{t-1};\mu_{\theta }\left(x_{t},t\right),\Sigma_{\theta }\left(x_{t},t\right)\right)$$

During the inverse process, our goal is to denoise the pure noise image $x_{T}$ and reconstruct the image that approximates the original $x_{0}$. That is, the noise removed in the reverse process is expected to approximate the noise added in the forward process. Therefore, this process can be transformed into predicting a noise $\epsilon_{\theta }\left(x_{t},t\right)$ to fit the noise ϵ added at time t. Thus, the optimization of the inverse process can be simplified as follows [19]:

(5) $$L_{DM}^{\mathrm{simple}\;}∶=E_{x_{0},\epsilon ∼N(0,1),t}\left[{∥\epsilon -\epsilon_{\theta }\left(x_{t},t\right)∥}_{2}^{2}\right]$$

Compared with DMs, LDMs undergo the diffusion process in a lower-dimensional latent space. As shown in Figure 1, the given real image $x_{0}$ is mapped from the pixel space to the latent space through an AutoEncoderKL ε and is represented as $z=\varepsilon \left(x_{0}\right)$,$\;z\in R^{h\times w\times 3}$. The reverse process can be simplified as the following formula [24]:

(6) $$L_{LDM}^{\mathrm{simple}\;}∶=E_{E(x_{0}),\epsilon ∼N(0,1),t}\left[{∥\epsilon -\epsilon_{\theta }\left(z_{t},t\right)∥}_{2}^{2}\right]$$

The optimization of the objective function is achieved by a time-conditional UNet. In this paper, in addition to the time condition, we also need to introduce EEG signals as the control condition y; that is, we also need to model the conditional distribution in the form of $p\left(z|y\right)$. This can be achieved by the conditional denoising autoencoder $\epsilon_{\theta }\left(z_{t},t,y\right)$. Based on the EEG–image pairs, we can train the model via the following formula:

(7) $$L_{LDM}:=E_{E(x_{0}),y,\epsilon ∼N(0,1),t}\left[{∥\epsilon -\epsilon_{\theta }\left(z_{t},t,\tau_{\theta }(y)\right)∥}_{2}^{2}\right]$$

where $\tau_{\theta }(y)$ is the FrozenCLIPEmbedder in Figure 1. The encoder converts the conditional EEG signal into an intermediate expression, which is then mapped to the middle layer of U-Net via cross-attention blocks. The formula for cross attention is described as:

(8) $$\mathrm{Attention}\left(Q,K,V\right)=\mathrm{softmax}\left(\frac{QK^{T}}{\sqrt{d}}\right)\cdot V$$

where $Q=W_{Q}^{\left(i\right)}\cdot \varphi_{i}\left(z_{t}\right)$, $K=W_{K}^{\left(i\right)}\cdot \tau_{\theta }(y)$, and $V=W_{V}^{\left(i\right)}\cdot \tau_{\theta }(y)$. In addition, $\varphi_{i}\left(z_{t}\right)$ is the intermediate representation when U-Net predicts $\epsilon_{\theta }.$ $W_{Q}^{\left(i\right)}$, and $W_{K}^{\left(i\right)}$ and $W_{V}^{\left(i\right)}$ are learnable projection matrices.

During the model fine-tuning process, we fix the remainder of the SD model and optimize the encoder $\tau_{\theta }(y)$, cross-attention head, and projection head at the same time. We used loss analysis based on Formula (7) to fine-tune the model. The encoder used in the SD model is a pretrained FrozenCLIPEmbedder. It is a text–image alignment model pretrained by the contrastive language–image pretraining (CLIP) [41] method. As shown in Figure 1, the EEG signal undergoes position embedding after passing through the feature extraction stage to match the text data. In this paper, EEG feature vectors that have undergone feature extraction and position encoding are used instead of text input to the pretrained FrozenCLIPEmbedder. The FrozenCLIPEmbedder was fine-tuned to help align the EEG feature vector space with the image feature space. Fine-tuning the cross-attention head is essential for bridging the pretraining conditional space and the latent space of the EEG features.

#### 2.2.3. Image Generation

Image generation is sampled from the standard normally distributed $x_{T}$. The reconstructed image corresponding to $x_{0}$ is calculated via the inverse diffusion process. In the image generation stage, as shown in Figure 1, the feature vectors obtained from feature extraction and position encoding are fed into the fine-tuned FrozenCLIPEmbedder to form an intermediate expression for input to U-Net. After the fine-tuning of the EEG–image pairs, the cross-attention head and projection head in U-Net learn the relationships between the EEG signals and image features. The alignment of the EEG feature space and image feature space is realized to some extent. Therefore, the feature vector output from the fine-tuned U-Net is regarded as the expression of the generated image in the latent space. AutoEncoderKL is used to decode the latent expression of the image and eventually recover the image of the pixel space. $\alpha_{t}=1-\beta_{t}$ and $\bar{\alpha_{t}}=\prod_{i=1}^{t}\alpha_{t}$ are defined using parametric renormalization. The mean value at time t is calculated by the following formula:

(9) $$\mu_{\theta }\left(x_{t},t\right)\;∶=\frac{1}{\sqrt{\alpha_{t}}}\left(x_{t}-\frac{1-\alpha_{t}}{\sqrt{1-\bar{\alpha_{t}}}}\epsilon_{\theta }\left(x_{t},t\right)\right)$$

The process of image generation is shown in Algorithm 1.
**Algorithm 1:** The Pipeline of the Image Generation Stage1: input EEG $x\in R^{1\times 128\times 440}$


2: do: Feature extraction and position embedding $\to x_{0}\in R^{77\times 768},x_{0}∽q\left(x_{0}\right)$
3: do: FrozenCLIPEmbedder $\to E(x_{0})$
4: for t=1⋯T, do:$q\left(x_{1}:x_{T}|E(x_{0}\right)\;∶=\prod_{t=1}^{T}q\left(x_{t}|x_{t-1}\right)\;\to x_{T}∽N(0,1)$
5: for t=T⋯1, z∽N(0,1) do:6: $x_{t-1}∶=\frac{1}{\sqrt{\alpha_{t}}}\left(x_{t}-\frac{1-\alpha_{t}}{\sqrt{1-\bar{\alpha_{t}}}}\epsilon_{\theta }\left(x_{t},t\right)\right)\to E(x_{0})\in R^{h\times w\times 3}$
7: do: AutoEncoderKL8: $return\;x_{0}\in R^{H\times W\times 3}$


As shown in Algorithm 1, the EEG feature generates the prompt representation $x_{0}\in R^{77\times 768}$ after feature extraction and position embedding. $x_{0}∽q\left(x_{0}\right)$ represents the data distribution corresponding to the real image. $x_{0}$ generates the expression $E(x_{0})$ of the potential space through the FrozenCLIPEmbedder. $x_{T}$ is formed through the forward noise addition process. Then, step 6 in Algorithm 1 is carried out to complete the image reconstruction of the potential space. According to step 6 and formula (9), we obtain $x_{t-1}=\mu_{\theta }\left(x_{t},t\right)$. Moreover, $\mu_{\theta }\left(x_{t},t\right)$ has completed the prediction in model fine-tuning and can directly use the model with updated parameters for image reconstruction. Then, the image is converted to pixel space through step 7. Therefore, the EEG-to-image task can be realized according to the algorithm in Algorithm 1.

## 3. Results

The proposed EEG-ConDiffusion framework was validated on an EEG-based visual classification dataset [12]. The visual stimuli in this dataset are derived from 40 classes of images in the ImageNet dataset [13]. The proposed framework combines EEG classification and image generation tasks, and we present the results of both tasks separately.

### 3.1. Results of EEG Classification

In the EEG classification experiments, we validated them separately on data within the ranges of 1–70 Hz and 5–95 Hz. The data were divided according to percentages of 70%, 15%, and 15%. The input shape of the EEG data is $X\in R^{b\times 1\times 128\times 440}$. Additionally, b represents the value of the batch size, which is designed to be 64. To train the model, we used the Adam optimizer to run 1000 epochs to minimize losses. In addition, the initial learning rate is configured as 0.01. Furthermore, in order to reduce computational resources and save model training time, our classification model training in the feature extraction stage and model fine-tuning in the image generation stage are conducted separately. The EEG data are first fed into our proposed ConvNet, and, after sufficient training, the model weights are saved. When generating images, we directly use the pretrained ConvNet to obtain highly discriminative category features.

#### 3.1.1. Evaluation Metrics for the EEG Classification Task

The classification accuracy along with kappa values were used to evaluate the classification efficiency of our proposed ConvNet. The accuracy is calculated as follows:

(10) $$accuracy=\frac{T_{P}+T_{N}}{T_{P}+F_{P}+F_{N}+T_{N}}$$

where ${\;T}_{P}$ and $F_{P}$ represent true positives and false positives, respectively. $T_{N}$ and $F_{N}$ represent true negatives and false negatives, respectively.

The formula for calculating kappa is as follows:

(11) $$kappa=\frac{P_{0}-P_{e}}{1-P_{e}}$$

$P_{0}$ represents the classification accuracy. $P_{e}$ represents a completely random rate of the classification accuracy. In the 40-category classification task in this paper, $P_{e}=0.025$.

#### 3.1.2. Classification Results Compared with the Baselines

To evaluate the feature extraction capability of the proposed EEGConvNet, we compared it with recent methods such as LSTM [12], EEGNet [16], and ChannelNet [1]. As shown in Table 1 and Table 2, we compared the performances of the proposed EEGConvNet and benchmark models on 5–95 Hz and 1–70 Hz data. We trained a single-person model for six subjects and evaluated the accuracy and kappa values of the EEGConvNet model and benchmark models. From Table 1 and Table 2, we can observe that the proposed EEGConvNet is verified on 5–95 Hz and 1–70 Hz data, and the average accuracy and average kappa value obtained are better than those of the benchmark models. The average accuracy of EEGConvNet was 67.97% on 5–95 Hz data, which was 56.34%, 30.60%, and 47.79% higher than that of LSTM [12], EEGNet [16], and ChannelNet [1], respectively. The average kappa value of EEGConvNet was 0.67, which was 0.58, 0.31, and 0.49 higher than that of LSTM [12], EEGNet [16], and ChannelNet [1], respectively. On the 1–70 Hz data, the average accuracy and average kappa value of EEGConvNet are 99.87% and 1.00, respectively, which are also better than those of the benchmark models. This indicates that the classification performance and model stability of EEGConvNet are good.

In addition, we conducted a Wilcoxon signed-rank test between the proposed EEGConvNet and the benchmark models in Table 3. EEGConvNet was tested with LSTM [12], EEGNet [16], and ChannelNet [1], and the *p* values obtained were 1, 2, and 3, respectively, all of which were less than 0.05. This shows that the improvement in the classification effectiveness of our proposed EEGConvNet model is statistically significant compared to that of the benchmark model.

Additionally, by comparing Table 1 and Table 2, we observe that the average accuracy and average kappa values obtained by the tested model in the signals of 1–70 Hz are better than those obtained at 5–95 Hz. We list the differences between the results in Table 4. The average accuracies of LSTM [12], EEGNet [16], ChannelNet [1], and EEGConvNet at 1–70 Hz are improved by 1%, 1%, 1%, and 1%, respectively, compared to those at 5–95 Hz. This shows that different frequencies have an important impact on the classification results of the model, and the data make it easier to extract and classify features at 1–70 Hz. Therefore, in the subsequent image generation stage, we use data at 1–70 Hz for model fine-tuning. Furthermore, the EEGConvNet model has achieved an average classification accuracy of 99.87% on data ranging from 1 to 70 Hz, demonstrating its ability to accurately classify EEG data. However, to further enhance classification accuracy, it is usually necessary to increase the depth and width of the network, which will increase the complexity and training time of the model. Therefore, EEGConvNet is already sufficiently and excellently suited for the feature extraction stage prior to classification tasks.

### 3.2. Results of Generating Images from EEG Signals

From the analysis in Section 2, it can be seen that the data with a frequency range of 1–70 Hz can be better extracted by the proposed EEGConvNet. In the image generation stage, we use the data extracted by EEGConvNet from 1 to 70 Hz as a condition and input a fine-tuned SD model to guide image generation. The images we need to reconstruct come from 40 categories of data from the ImageNet [13] dataset. To evaluate the image generation of the EEGConDiffusion framework, we generated three predicted images for each category for comparison with the real image. When fine-tuning the model, we trained the SD model with 500 epochs using a learning rate of 5.3 × 10^−5^. Due to limited laboratory conditions, we trained with a GPU model RTX 3090 (24 GB) from Intel Corporation in the United States with a batch size set to three. The total training time for each subject is approximately 58 h. We train six individual models in parallel using six servers of the same model. In addition, the number of sampling steps for the SD model is set to 250. In the image generation stage, the number of sampling steps for the SD model is also 250.

#### 3.2.1. Evaluation Metrics for the Image Generation Tasks

To assess the quality of image generation, we used the Fréchet inception distance (FID) [42], inception score (IS) [43,44], and top-k [45] classification task accuracy as evaluation metrics. FID evaluates the differences between the generated image and the ground truth image by measuring the distance between their feature levels. Its mathematical description is as follows:

(12) $$FID={\left\|\mu_{r-}\mu_{g}\right\|}^{2}+T_{r}\left(\sum_{r}+\sum_{g}-2{\left(\sum_{r}\sum_{g}\right)}^{1/2}\right)$$

where $\mu_{r}\;$and $\mu_{g}$ represent the feature means of the authentic image and the reconstructed image, respectively. $\sum_{r}$ and $\sum_{g}$ represent the covariance matrix of the real picture and the generated picture, respectively. $T_{r}$ represents the trace. The IS is used to evaluate the clarity and diversity of image generation. It uses the pretrained Inception model [44] to perform category discrimination and calculates the authenticity score and diversity score of the generated images. Its mathematical expression is:

(13) $$IS=exp\left(E_{x\sim p_{g}}D_{KL}\left(P\left(y|x\right)\left\|P\left(y\right)\right.\right)\right)$$

where $E_{x\sim p_{g}}$ represents the average value of the traversed images. $D_{KL}$ stands for KL divergence. $P\left(y|x\right)$ and $P\left(y\right)$ represent the conditional distribution and edge probability distribution of the image category, respectively. The top-k classification task is used to evaluate the category accuracy of image generation. In this paper, we use the pretrained ImageNet1K [45] model to evaluate the classification accuracy of the 50-way top-1 and top-5 classification accuracies of the generated images.

#### 3.2.2. Ablation Experiments

Since the training and fine-tuning of the SD model require considerable time and computational costs, we need to determine the conditions for the input of the SD model before the fine-tuning stage. To this end, we performed four types of ablation experiments before fine-tuning the SD model. They are described as follows:

- The EEG data were used directly. The data shape is changed to (77,768) through a linear layer and a conv1d layer and then imported into a fixed SD model;
- Position embedding was performed on the EEG data. The input shape is then changed to (77,768) through the linear and conv1d layers, and the fixed SD model is then entered;
- The pretrained EEGConvNet model was used to extract features from the EEG data. The features output by the classification model, with a shape of (77,768), are then used as input to the pretrained SD model;
- After extracting the EEG features using the pretrained EEGConvNet model, position embedding was performed on the EEG data. The shape features (77,768) are then fed into the pretrained SD model.

As shown in Table 5, we evaluated the image generation performance under four different experiments using FID. We expect the generated image to have a smaller FID value. From the FID results, we can see that the average FIDs for EEGs with shape adaptation are 243.88 and 243.03 for position embedding. Position embedding slightly improves the generation effect of images. The FID after feature extraction is 46.37, which is quite different from the result without feature extraction. This shows that EEG feature extraction through EEGConvNet plays an important role in improving image generation performance. Therefore, we finally use EEG features that have undergone feature extraction and positional embedding as the guiding conditions for the SD model.

#### 3.2.3. Image Generation Results Compared with the Baselines

**Figure 3:**
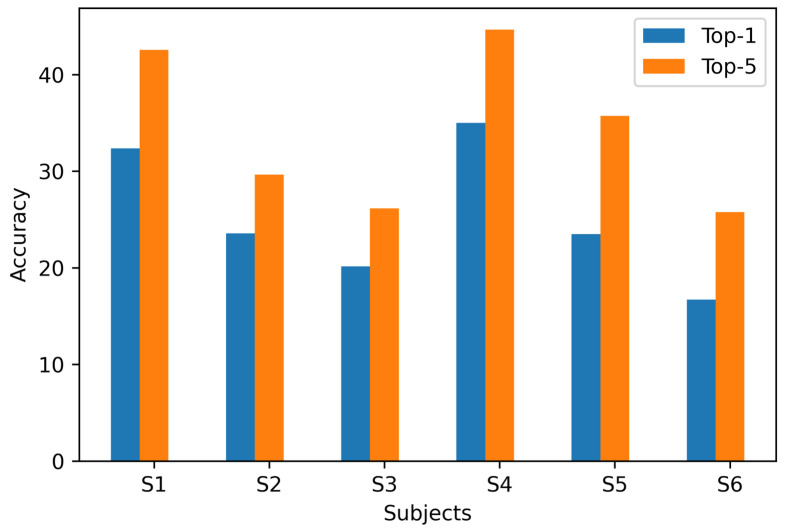
The top-1 and top-5 classification results of the proposed EEG-ConDiffusion framework.

To test the image generation quality of our EEG-ConDiffusion framework, we compared it with five methods developed in recent years. Kavasidis et al. [25] combined LSTM feature extraction and conditional GAN to improve the image generation effect and verified the effect of VAE and GAN for image generation. Zheng et al. [27] combined feature extraction and the SNGAN to generate images. Khare et al. [29] proposed a NeuroVision framework for image generation in combination with conditional ProGAN (cProGAN). We used the IS to evaluate the image generation performance by comparing the proposed EEG-ConDiffusion framework with the four baseline models. The higher the value of IS, the better the image is generated. In addition, we used the top-1 and top-5 tasks to verify the classification accuracy of the images generated by the proposed EEG-ConDiffusion framework. The IS and top-k classification results of the proposed EEG-ConDiffusion framework are shown in Table 6. In addition, as shown in Figure 3, the classification accuracy of the EEG-ConDiffusion framework on the top-1 and top-5 classification tasks is visualized.

Table 7 shows the average IS results of the EEG-ConDiffusion framework and the baselines. The IS result obtained by our EEG-ConDiffusion method is 12.38, which is 7.89, 7.31, 6.85, and 7.23 higher than that of VAE [25], GAN [25], SNGAN [27], and cProGAN [29], respectively. We can see that the IS results of GAN-based image generation methods are better than those of VAE. Our EEG-ConDiffusion IS results are better than those of all baseline models, and the improvement effect is obvious. This shows the powerful ability of pretrained SD models to generate images from EEG signals.

**Figure 4:**
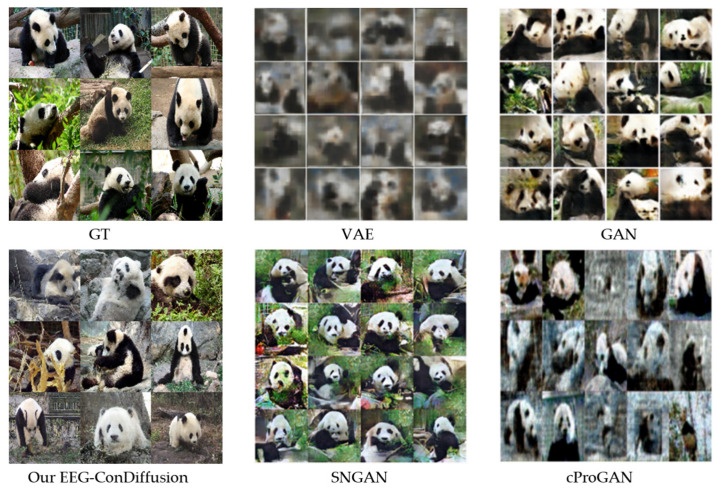
Comparison of the proposed EEG-ConDiffusion framework and benchmark models for generating images of pandas. This figure includes images generated with VAE [25], GAN [25], SNGAN [27], cProGAN [29], and our EEG-ConDiffusion, as well as GT images. GT represents the ground truth images.

Figure 4 shows the images of pandas generated by our EEG ConDiffusion and benchmark models. The images generated using the VAE method are the most blurry. The images generated using our EEG-ConDiffusion method not only have higher clarity than the benchmark model but also have higher realism.

**Figure 5:**
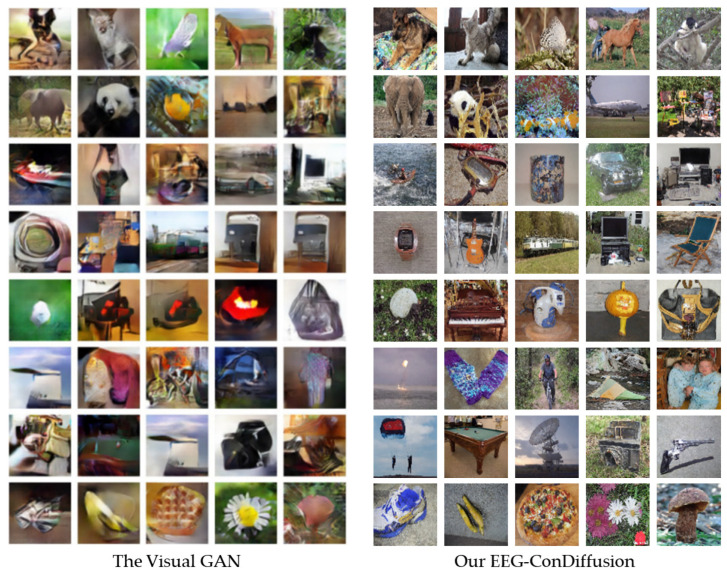
Comparison between the images generated by our EEG ConDiffusion and the Visual GAN [46].

In addition, we compared our EEG-ConDiffusion framework with Visual GAN [46]. Shimizu et al. proposed a Sinc-EEGNet model to extract features of brain signals. The Sinc-EEGNet model combines a CNN model and attention mechanism, and its classification accuracy is 45%. Then, the Sinc-EEGNet model was combined with the GAN model for image generation. The average classification accuracy of the images generated with the test set is 18.4%. As can be seen from Table 2 and Table 6, the average classification accuracy of our EEG-ConDiffusion framework is 99.87% in the feature extraction stage, and the average classification accuracy of the top-1 task in the image generation stage is 25.21%. As shown in Figure 5, renderings of 40 category images were generated for the EEG-ConDiffusion framework and the Visual GAN [46] model. We can see that the images generated by the EEG-ConDiffusion framework are sharper and more realistic.

**Figure 6:**
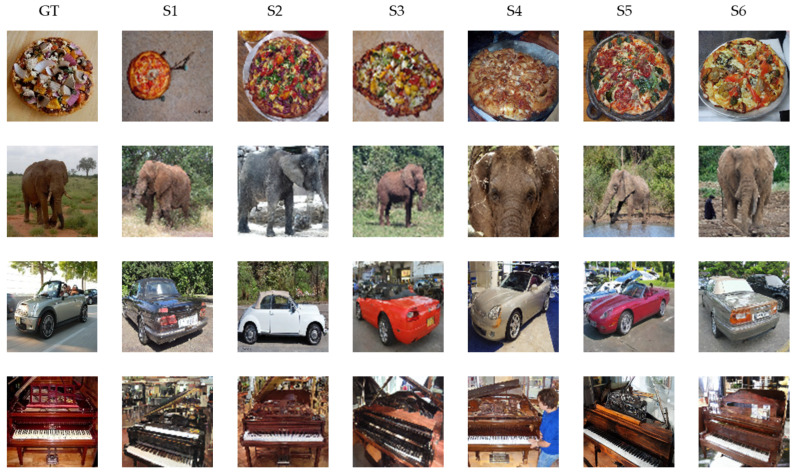
Comparison between the images generated by our EEG ConDiffusion and the ground truth. S1, S2, S3, S4, S5, and S6 represent the 6 subjects. GT represents the ground truth.

To further demonstrate the generation effect of our EEG-Diffusion method, we present the generated images of pizzas, elephants, cars, and pianos in Figure 6 and compare them with the ground truth images.

**Figure 7:**
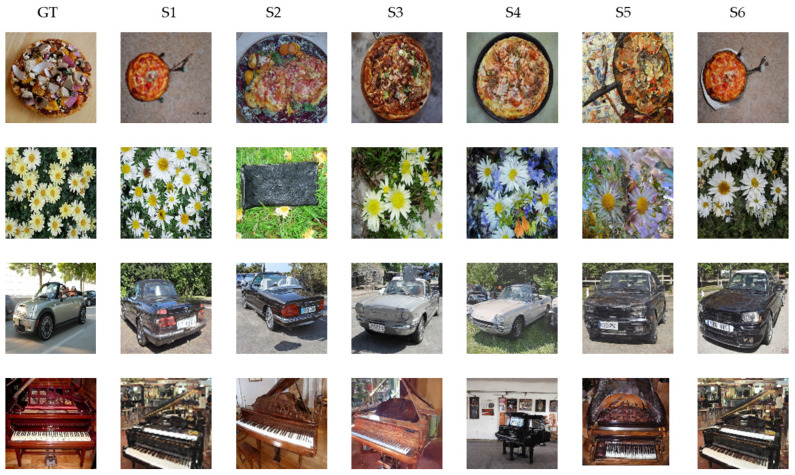
Comparison between the images generated by our EEG ConDiffusion and the ground truth in the inter-subject transfer learning experiment. S1, S2, S3, S4, S5, and S6 represent the 6 subjects. GT represents the ground truth.

The above discussions are based on intra-subject experiments. In order to evaluate the generalization performance of our proposed model. We used the model of subject 1 with the highest IS as the optimal model. The model was used to combine the test set data of other subjects to generate corresponding images. As shown in Table 8, we calculated the IS of S2~S6, and the average IS result was 11.74, which was better than the four benchmark models. As shown in Figure 7, the images generated by model migration are of high quality. Thus, the generalization performance of our EEG-ConDiffusion framework is demonstrated.

## 4. Discussion

In this work, we propose a framework called EEG-ConDiffusion that combines feature extraction and pretrained conditional diffusion to generate images from EEG signals. We divide the EEG-ConDiffusion framework into three stages: feature extraction, SD model fine-tuning, and image generation.

In the feature extraction stage, we propose an EEGConvNet to perform feature extraction on EEG signals. The output EEG features are reshaped and positionally embedded to accommodate the input of the SD model. We validated the classification performance of the EEGConvNet and baselines on EEG data at 5–95 Hz and 1–70 Hz, respectively. The EEG data are derived from the EEG-based visual classification dataset [12]. As shown in Table 4, all the tested models achieved better results on the 1–70 Hz data than on the 5–95 Hz data. This shows that the data in the dataset with a filter range of 1–70 Hz can be better extracted for features. Therefore, in the subsequent SD model fine-tuning and image generation stages, we use data with a frequency range of 1–70 Hz. In addition, Table 1 shows that the average accuracy of EEGConvNet is 67.97%, which is 56.34%, 47.79%, and 30.60% higher than that of LSTM [12], ChannelNet [1], and EEGNet [16], respectively. As shown in Table 2, the average accuracy of EEGConvNet is 99.87%, which is 38.55%, 6.26%, and 0.79% higher than that of LSTM [12], ChannelNet [1], and EEGNet [16], respectively. When EEGConvNet is validated in both frequency ranges, the average kappa values are also greater than those of the baseline models. In addition, as shown in Table 3, we also performed a Wilcoxon signed-rank test on the results between the EEGConvNet and the baseline models. The obtained *p* values were all less than 0.05, which proved the statistical significance of EEGConvNet.

In the model fine-tuning stage, we use the EEG features that have gone through the process of feature extraction, shape adaptation, and position embedding as the conditions of the SD model to train the model. The SD model is a pretrained text-to-image model based on LDMs [24]. LDMs transform the diffusion process of DMs [19] into a low-dimensional latent space, which reduces the computational complexity and cost. This allows us to fine-tune the SD model with just a single RTX3090 GPU. In addition, LDMs introduce cross-attention in U-Net, thus enabling multimodal transitions. This is also the key to the realization of text-to-image in the SD model. This inspired us to use LDMs to implement EEG-to-image tasks. However, training LDMs from the start to the finish requires a large computational cost, long computation time, and problems such as schema crashes during training. To overcome this difficulty, we used a pretrained SD model, which has gained great attention in the text-to-image field. Only small EEG–image data pairs are needed to fine-tune the SD model to realize the consistency of image and EEG features.

In the image generation stage, we send the EEG features that have gone through the process of feature extraction, shape change, and position embedding into the cross-attention module of the fine-tuned SD model. To validate the performance of the images generated by our EEG-Condiffusion framework, we tested them on the EEG-based visual classification dataset [12] and the ImageNet dataset [13]. As shown in Table 5, we performed ablation experiments for different EEG preprocessing methods and evaluated them with FID results. The results show that the FID of the EEG input obtained through EEGConvNet and position embedding is 41.62, which is 202.26, 201.41, and 4.75 lower than that obtained through shape adaptation only, shape adaptation, and position embedding and feature extraction only, respectively. The process of position embedding slightly reduces the FID results. The feature extraction process has a significant impact on the FID results. This suggests that feature extraction plays an important role in image generation. As shown in Table 6 and Table 7, we show the image generation results of the EEG-ConvDiffusion framework and benchmark models and evaluate them with the IS results. The results show that the average IS of the EEG-ConDiffusion framework is 12.38, which is 7.89, 7.31, 6.85, and 7.23 higher than those of the VAE [25], GAN [25], SNGAN [27], and cProGAN [29], respectively. In addition, as shown in Figure 3, we show the accuracy of the top-1 and top-5 classification tasks for images generated by the model for each subject based on the EEG-ConDiffusion framework. The results show that this method can guarantee the authenticity of image generation to a certain extent. Figure 4 shows the panda images generated by our EEG-ConDiffusion framework and benchmark models and compares them with the ground truth images. The results show that the images generated by the VAE [25] method are the fuzziest, while the clarity of the images generated by the GAN [25], SNGAN [27], and cProGAN [29] methods is improved, but the generated images lack authenticity. The images generated by our EEG-ConDiffusion method are improved in terms of clarity and image authenticity. In addition, we compare the classification accuracy of the proposed framework with Visual GAN [46] in the feature extraction and generation stages. This proves that our EEG-ConDiffusion framework improves the quality of image generation with effective EEG features. In addition, as shown in Table 8, we performed model transfer experiments between subjects based on the model of subject 1 and obtained high-quality generated images. The generalization performance of our proposed EEG-ConDiffusion framework is demonstrated. To further demonstrate the generative performance of our EEG-ConDiffusion framework, we show the images of pizzas, elephants, cars, and pianos generated by the framework and the corresponding ground truth images in Figure 5. The images generated by each single-person model are similar to the ground truth images.

## 5. Conclusions

In summary, we combined EEG feature extraction and image generation tasks to construct an EEG-ConDiffusion framework for generating images from EEG signals. Our work explored the feasibility of using multimodal large models to study EEG signals. The EEG-ConDiffusion framework uses EEGConvNet to extract highly discriminating semantic information from EEG signals and then uses the LDM-based SD model to generate high-resolution images. We evaluated the feature extraction stage and the image generation stage and revealed the powerful feature extraction ability and image generation performance of the EEG-ConDiffusion framework.

In future work, we will make more efforts in EEG–image feature matching. Additionally, the use of multi-level EEG features to match image features proposed by Shen et al. [47] is a very good inspiration. In addition, we will optimize the inter-subject transfer experiments to enhance the model’s generalizability.

## Figures and Tables

**Table 1:** Accuracy and kappa values of the EEG classification task on the EEG-based visual classification dataset. The frequency range of the EEG data is 5–95 Hz.

Subjects	Method							
	LSTM [12]		EEGNet [16]		ChannelNet [1]		EEGConvNet	
	Acc (%)	Kappa	Acc (%)	Kappa	Acc (%)	Kappa	Acc (%)	Kappa
S1	7.03	0.04	18.75	0.17	5.47	0.03	56.25	0.55
S2	9.38	0.07	54.69	0.53	26.56	0.25	79.69	0.79
S3	8.81	0.05	50.78	0.49	25.78	0.24	67.97	0.68
S4	4.69	0.02	32.81	0.31	25.78	0.24	78.12	0.77
S5	23.43	0.22	35.94	0.34	25.00	0.23	64.84	0.64
S6	16.41	0.14	31.25	0.30	12.50	0.10	60.94	0.60
Mean	11.63	0.09	37.37	0.36	20.18	0.18	67.97	0.67

**Table 2:** Accuracy and kappa values of the EEG classification task on the EEG-based visual classification dataset. The frequency range of the EEG data is 1–70 Hz.

Subjects	Method							
	LSTM [12]		EEGNet [16]		ChannelNet [1]		EEGConvNet	
	Acc (%)	Kappa	Acc (%)	Kappa	Acc (%)	Kappa	Acc (%)	Kappa
S1	75.78	0.75	98.43	0.98	93.75	0.93	100.00	1.00
S2	53.12	0.51	98.43	0.98	85.93	0.85	99.22	0.99
S3	61.71	0.61	100.00	1.00	96.87	0.97	100.00	1.00
S4	73.43	0.72	99.21	0.99	99.21	0.99	100.00	1.00
S5	58.59	0.57	99.21	0.99	93.75	0.93	100.00	1.00
S6	45.31	0.44	99.21	0.99	92.18	0.92	100.00	1.00
Mean	61.32	0.60	99.08	0.98	93.61	0.93	99.87	1.00

**Table 3:** Wilcoxon signed-rank test between the proposed EEGConvNet and the baselines.

Frequencies	Method					
	LSTM [12]		EEGNet [16]		ChannelNet [1]	
	P-Acc (%)	P-Kappa	P-Acc (%)	P-Kappa	P-Acc (%)	P-Kappa
5–95 Hz	0.016	0.016	0.016	0.016	0.016	0.016
1–70 Hz	0.016	0.016	0.027	0.024	0.016	0.016

**Table 4:** Differences in the classification performances of EEGConvNet and the baselines on 1–70 Hz and 5–95 Hz data.

Frequencies	Method							
	LSTM [12]		EEGNet [16]		ChannelNet [1]		EEGConvNet	
	Acc (%)	Kappa	Acc (%)	Kappa	Acc (%)	Kappa	Acc (%)	Kappa
5–95 Hz	11.63	0.54	37.37	0.36	20.18	0.18	67.97	0.67
1–70 Hz	61.32	0.60	99.08	0.98	93.61	0.93	99.06	0.99
Difference	49.69	0.06	61.71	0.62	73.43	0.75	31.09	0.32

**Table 5:** FID results of four ablation experiments and the proposed EEG-ConDiffusion framework.

Subjects	Ablation Experiments			
	EEG with Shape Adaptation	EEG with Shape Adaptation and Position Embedding	EEG with Feature Extraction	EEG with Feature Extraction and Position Embedding
	FID	FID	FID	FID
S1	239.48	240.99	56.10	47.64
S2	246.41	246.04	42.84	34.78
S3	245.48	239.08	57.23	42.43
S4	242.57	246.44	48.98	33.73
S5	246.18	241.82	47.16	42.20
S6	243.13	243.80	25.89	48.92
Mean	243.88	243.03	46.37	41.62

**Table 6:** The IS and top-k classification results of the proposed EEG-ConDiffusion framework.

Subjects	Metrics		
	IS	Top-1 Acc (%)	Top-5 Acc (%)
S1	12.86	32.36	42.56
S2	11.95	23.56	29.64
S3	12.49	20.16	26.16
S4	12.47	35.00	44.64
S5	12.46	23.48	35.72
S6	12.03	16.72	25.76
Mean	12.38	25.21	34.08

**Table 7:** The IS results of the EEG-ConDiffusion framework and baseline models.

Metric	Method				
	Kavasidis et al. VAE [25]	Kavasidis et al. GAN [25]	Zheng et al. SNGAN [27]	Khare et al. cProGAN [29]	Our EEG-ConDiffusion
IS	4.49	5.07	5.53	5.15	12.38

**Table 8:** The IS results of the EEG-ConDiffusion framework in the inter-subject transfer learning experiment.

Subjects	S1	S2	S3	S4	S5	S6	Mean
IS	12.86	11.73	11.57	12.12	10.49	11.66	11.74

## Data Availability

The data used in this article can be accessed at https://tinyurl.com/eeg-visual-classification (accessed on 8 September 2023). And the code is at https://github.com/ perceivelab/eeg_visual_classification (accessed on 8 September 2023).
